# Intercropping changed the soil microbial community composition but no significant effect on alpha diversity

**DOI:** 10.3389/fmicb.2024.1370996

**Published:** 2024-03-20

**Authors:** Jiaying Liu, Weixi Zhang, Chao Teng, Zhongyi Pang, Yanhui Peng, Jian Qiu, Jiawei Lei, Xiaohua Su, Wenxu Zhu, Changjun Ding

**Affiliations:** ^1^College of Forestry, Shenyang Agricultural University, Shenyang, China; ^2^State Key Laboratory of Tree Genetics and Breeding, Research Institute of Forestry, Chinese Academy of Forestry, Beijing, China; ^3^Key Laboratory of Tree Breeding and Cultivation of State Forestry Administration, Research Institute of Forestry, Chinese Academy of Forestry, Beijing, China; ^4^Liaoning Non-Ferrous Geological Exploration and Research Institute Co., Ltd., Shenyang, China; ^5^Xinmin Machinery Forest Farm, Xinmin, China; ^6^State-owned Xinbin Manchu Autonomous County Douling Forest Farm, Fushun, China

**Keywords:** intercropping, soil bacterial and fungal communities, agroforestry intercropping, *Glycine max*, *Populus cathayana* × *candansis* cv. *Xinlin No. 1*

## Abstract

**Introduction:**

Enhancing the planning of the forest-agricultural composite model and increasing the efficiency with which forest land is utilized could benefit from a thorough understanding of the impacts of intercropping between forests and agriculture on soil physicochemical properties and microbial communities.

**Methods:**

*Populus cathayana* × *candansis* cv. *Xinlin No.1* and *Glycine max* intercrop soils, along with their corresponding monocrops, were used in this study’s llumina high-throughput sequencing analysis to determine the composition and diversity of soil bacterial and fungal communities.

**Results:**

The findings indicated that intercropping considerably raised the soil’s total phosphorus content and significantly lowered the soil’s carbon nitrogen ratio when compared to poplar single cropping. Furthermore, the total carbon and nitrogen content of soil was increased and the soil pH was decreased. The sequencing results showed that intercropping had no significant effect on soil alpha diversity. Intercropping could increase the composition of fungal community and decrease the composition of bacterial community in poplar soil. At the phylum level, intercropping significantly increased the relative abundance of four dominant phyla, i.e., Patescibacteria, Proteobacteria, Patescibacteria and Deinococcus-Thermus. And the relative abundances of only two dominant phyla were significantly increased. It was found that soil total phosphorus and available phosphorus content had the strongest correlation with soil bacterial community diversity, and soil pH had the strongest correlation with soil fungal community diversity.

**Discussion:**

The results of this study were similar to those of previous studies. This study can serve as a theoretical foundation for the development of a poplar and black bean-based forest-agricultural complex management system in the future.

## Introduction

An essential part of the terrestrial ecosystem is the soil (Staddon, 2004). The primary factor in sustaining soil health is the functioning of the soil life system, which is fueled by the ecological, environmental, and immune roles played by soil microorganisms. It can control the functional diversity of the soil and take part in the carbon cycle of the ecosystem. In addition, it has the ability to break down plant residues, maintain soil fertility, and improve soil nutrients (van Agtmaal et al., 2018; Gilmullina et al., 2020; Wang et al., 2020; Cuartero et al., 2022; Fan et al., 2022). When assessing the degree of soil health, the biomass, abundance and activity of soil microorganisms are crucial guiding factors (Rodgers et al., 2021). Soil microbial community activities affect soil fertility and play an important role in organic matter, humus and soil nutrient cycling and conversion (Kirk et al., 2004). As a result, the importance of soil microorganisms in the soil ecosystem has received increasing attention (Neira et al., 2021). The abundance and diversity of different types of soil microorganisms can affect soil physical and chemical properties (Christel et al., 2023).

Intercropping is a productive cultivation technique that is widely used in agro-ecosystems worldwide (Rezaei-Chiyaneh et al., 2021). It can preserve the quality of the soil, increase crop productivity, and efficiently use environmental resources like light, water and fertilizer (Kang et al., 2021; Cuartero et al., 2022). Because the species in agroforestry intercropping complement each other, plant yield and resource utilization efficiency are increased, resulting in more full utilization of light, heat and water. When compared to a pure forest, the management of forest compounds can lower soil moisture evaporation, enhance soil condition, and preserve soil fertility (Fleskens et al., 2009). Through the effective use of shady space to develop under forest industry according to local conditions, it can effectively improve the comprehensive benefits of forest land, increase the added value of forestry, expand the development space of rural industry, achieve a win-win situation of farmers’ income increase and ecological stability, and help rural revitalization (Hong et al., 2017).

Numerous previous studies have demonstrated that intercropping practices can dramatically change the amount of nutrients in soil (Razavi et al., 2016; Gómez-Sagasti et al., 2021; Young et al., 2021). For instance, nitrogen-fixing crops fix atmospheric nitrogen through rhizobia when intercropping with other non-nitrogen-fixing tree species. This can supply additional nutrients for non-nitrogen-fixing tree species on top of their own nutrient needs. Intercropping in the change of soil physical and chemical properties at the same time, still can cause the change of soil microbial community, further influence soil health and quality (Fu et al., 2019). The structure of the soil microbial community varies significantly between intercropping and monocropping, according to earlier research (Cuartero et al., 2022). Based on the soil microbial community quantity, intercropping microorganisms were generally higher than that of single cropping (Li et al., 2018). However, the effect of tree-based intercropping on soil microbial diversity is controversial, and it does not increase bacterial or fungal diversity in all cases (Lacombe et al., 2009; Bainard et al., 2011, 2013). For example, Bardhan et al. (2013) studied the composition and diversity of soil bacterial communities in *Acer saccharinum* agroforestry systems and found no difference in the composition or diversity of soil bacteria between trees and crop rows. Banerjee et al. (2016) found that temperate agroforestry did not promote soil bacterial diversity, but increased bacterial abundance.

Poplar is one of the fastest growing timber species and has the largest planted area in the world’s mid-latitude plains (Hou et al., 2019). In 2016, the IPC (International Commission on Poplar and Other Fast-growing Trees that Sustain People and the Environment) reported that the total area of poplar cultivation worldwide had increased to 31.4 million hm^2^ (Lu et al., 2022). A large number of poplar forests are planted in the state-owned mechanical forest farm in Xinmin City, Liaoning Province, China. Intercropping under these forests can make full use of the local soil area. At present, the hybrid model of poplar intercropping forest has become one of the main planting models in this area. Intercropping of legumes with trees is a particularly sustainable and beneficial agricultural practice because nitrogen-fixing crops provide natural nitrogen fertilizer for tree growth (Peng et al., 2009; Rivest et al., 2010; Li et al., 2016). And it can also improve the growth of the legume crop because it can make it more competitive for nitrogen in the soil, forcing the legume to fix more N_2_ from the atmosphere (Hauggaard-Nielsen et al., 2001; Neumann et al., 2007). However, the effect of this intercropping pattern on soil microbial communities on there is still unclear. So, in this study, *Populus cathayana* × *candansis* cv. *Xinlin No. 1* and *Glycine max* were used as research objects to analyze and compare the changes of soil nutrients and soil microbial community composition and diversity after intercropping. We hypothesized that (1) after intercropping, soil nutrients would change significantly, especially the soil total nitrogen content of intercropping would increase significantly compared with poplar single cropping; (2) the composition of soil microbial community changed after intercropping, but there were significant differences in community diversity; and (3) changes in soil microbial community composition and diversity were related to changes in soil nutrient content.

## Materials and methods

### Field experiment design and soil sample collection

Field sampling and random block design were used to sample 0–10 cm soil from poplar single cropping (PC), black bean single cropping (GM) and poplar black bean intercropping (PG) in Xinmin Mechanical Forest Farm (located in Xinmin City, Liaoning Province, China; 41°59′59″ N, 122°48′19″ E) in August 2022. In order to avoid the interaction of two similar models and the stand edge effect, the quadrat of each model avoided the stand edge area. The poplar was “*Populus cathayana* × *candansis* cv. *Xinlin No. 1*” cultivated by state-owned Xinmin Mechanical Forest Farm, and the variety of black bean (*Glycine max*) was “green kernel black bean.” Three repeated quadrats were randomly selected for each model, and the soil samples of each quadrat were divided into two parts. The obtained soil was immediately transported back to the laboratory in an ice box. A part of soil samples was stored at −80°C to extract DNA and determine the composition and structure of soil microbial community. The other soil samples were dried in a ventilated and dry room, then ground and passed through a 100-mesh screen for the determination of soil chemical properties.

### Soil chemical properties determination

The pH value of soil was measured by potentiometer, and the ratio of soil to water was 1:2.5. The elemental analyzer (Germany’s Elementar Vario EL III) measured the total carbon and total nitrogen contents of the soil (Song et al., 2015). The molybdenum antimony anti-colorimetric method was used to determine the total phosphorus content of the soil (Ba et al., 2020). By using the NaHCO_3_ leaching-molybdenum antimony anti-colorimetric method, soil available phosphorus was calculated (Huang et al., 2020).

### DNA extraction and high-throughput sequencing

The OMEGA Soil DNA Kit (Omega Bio-Tek, Norcross, GA, United States) was used to extract total DNA from the samples. Thermo Fisher Scientific’s NanoDrop NC2000 spectrophotometer and agarose gel electrophoresis for extracted DNA (agarose concentration of 1.2%) were used to measure the quantity and quality of DNA. With the assistance of the primers 338F (5′-ACTCCTACGGGAGGCAGCA-3′) and 806R (5′-GGACTACHVGGGTWTCTAAT-3′), the 16S V3_V4 region of the soil bacterium was amplified (Claesson et al., 2009). Additionally, the fungal ITS V1 region was amplified using primers ITS5 (5′-GGAAGTAAAAGTCGTAACAAGG-3′) and ITS2 (5′-GCTGCGTTCTTCATCGATGC-3′) (White et al., 1990). Total volume for the PCR system is 25 μL, which includes 5 μL of reaction buffer, 5 μL of GC buffer, 2 μL of dNTP (2.5 mM), 1 μL of forward primer, and 1 μL of re-verse primer. A total of 2 μL of DNA template, 8.75 μL of ddH_2_O, and 0.25 μL of Q5 DNA polymerase. Initial denaturation at 98°C for 2 min, followed by 98°C for 15 s, 55°C for 30 s, and 72°C for 30 s for 25 cycles, and a final extension at 72°C for 5 min, 10°C hold for 25 cycles, were the amplification parameters. The Quant-iT PicoGreen dsDNA Assay Kit (Invitrogen, Carlsbad, CA, United States) was used to quantify the PCR amplicons after they had been purified using Vazyme VAHTSTM DNA Clean Beads (Vazyme, Nanjing, China). Shanghai Personal Biotechnology Co., Ltd. used the Illlumina NovaSeq platform and NovaSeq 6000 SP Reagent Kit (500 cycles) to perform 250 pair-end sequencing. Following the individual quantification step, amplicons were pooled in equal amounts. The raw reads were deposited in the NCBI SRA database with accession number PRJNA1007870.

### Sequence analysis

According to the official tutorials,^1^ microbiome bioinformatics were carried out using QIIME 2 (2019.4) with a minor modification (Bolyen et al., 2019). In a nutshell, primers were cut with the cutadapt plugin after raw sequence data were demultiplexed using the demux plugin. The DADA2 plugin was then used to quality filter, denoise, merge, and remove chimera from the sequences (Callahan et al., 2016). Using fasttree2 and mafft, non-singleton amplicon sequence variants (ASVs) were used to create a phylogenetic tree (Katoh, 2002; Price et al., 2009). Alpha-diversity metrics [Observed species richness (Liu et al., 2022), Simpson index (Simpson, 1949), and Pielou’s evenness index (Pielou, 1966)], beta diversity metrics (Bray-Curtis dissimilarity) were estimated using the diversity plugin. The SILVA Release 132 Database (Bacterium) and UNITE Release 8.0 Database (Fungus) were used as the sources for the naive Bayes taxonomy classifier in the feature-classifier plugin to assign taxonomy to ASVs (Kõljalg et al., 2013; Bokulich et al., 2018).

### Data analytics

The main tools used to analyze sequence data were QIIME2 and R packages (v3.2.0). Using the ASV table in QIIME2, ASV-level alpha diversity indices, including the Observed species richness, Simpson index, and Pielou’s evenness index, were calculated and visualized as box plots (Simpson, 1949; Pielou, 1966; Liu et al., 2022). The data in the table above were plotted as box plots with the help of QIIME2 (2019.4) and the ggplot2 package for the R package (v3.2.0) to visualize the differences in alpha diversity between the various specimen groups (Wickham, 2016). The significance of the differences could be confirmed using the Kruskal-Wallis’s rank sum test and the dunn’test as a *post-hoc* test. Based on the occurrence of ASVs across samples/groups regardless of their relative abundance, a Venn diagram was created to visualize the shared and unique ASVs among samples or groups using the R package “VennDiagram” (Zaura et al., 2009). The significant difference in soil chemical properties was examined using one-way ANOVA, and the post-test was carried out using the S-N-K *q*-test. IBM SPSS Statistics 26.0 was used to analyze data from significant differences in soil chemical properties processed by Excel (2019). The information in the table demonstrated the standard deviations of repeated averages. After removing singletons from the feature list, the QIIME2 (2019.4) “qiime taxa barplot” was used to visualize the compositional distribution of each sample at the four taxonomic levels of phylum, and genus. Relying on R’s pheatmap package, heatmaps were created using abundance data from the genera in the top 20 of average abundance. Redundancy analysis (RDA) using Canoco 5 was used to examine the relationships between soil environmental factors and microorganic community composition. STAMP software was used for difference analysis to compare the phylum level differences of different microbial communities (Parks et al., 2014).

## Results

### Chemical properties of soil under different treatments

There were differences in pH value, total carbon, total nitrogen, total phosphorus and available phosphorus contents and carbon nitrogen ratio of soil among different samples (Table 1). The contents of soil total phosphorus and available phosphorus in PG were significantly higher than those in PC (*p* < 0.05). Soil pH value (*p* > 0.05) and C/N (*p* < 0.05) were lower in PG than in PC and GM. PC had the highest pH value. The total carbon, total nitrogen and carbon nitrogen ratio of GM were the highest. The total carbon and total nitrogen of PG were higher than PC and lower than GM.

**TABLE 1:** Chemical properties of soil.

	pH value	Total carbon (g⋅kg^–1^)	Total nitrogen (g⋅kg^–1^)	Carbon nitrogen ratio	Total phosphorus (g⋅kg^–1^)	Available phosphorus (mg⋅kg^–1^)
PC	7.79 ± 0.17A	4.05 ± 0.05B	0.48 ± 0.01B	8.43 ± 0.11B	0.15 ± 0.01B	15.69 ± 2.15B
GM	7.45 ± 0.15A	6.81 ± 0.41A	0.72 ± 0.05A	9.53 ± 0.17A	0.23 ± 0.01A	19.85 ± 1.60B
PG	7.22 ± 0.10A	4.24 ± 0.29B	0.57 ± 0.03B	7.47 ± 0.17C	0.24 ± 0.02A	34.39 ± 2.72A
*F*-value	4.036	28.292	11.060	46.018	19.162	19.908
*p-*value	0.078	0.001	0.010	2.29 × 10^–4^	0.002	0.002

The data in the table are average ± standard error. Different capital letters indicate a significant difference at *p* < 0.05. PC, poplar single cropping; GM, black bean single cropping; PG, poplar black bean intercropping.

### Composition and diversity of soil microbial communities under different treatments

DADA2 analysis is equivalent to clustering with 100% similarity. With the increase of the drawing depth, the rarefaction curve has flattened, indicating that the existing sequencing results have been sufficient to reflect the microbial diversity contained in the current sample (Supplementary Figure 1).

There was no significant difference in alpha diversity in both bacterial and fungal communities (*p* > 0.05; Table 2). The Simpson index and Pielou’s evenness index of PG microbe (bacteria and fungi) were the highest (bacterial averages of 0.999 and 0.908, respectively; fungal averages of 0.977 and 0.772, respectively), while the PC community had the lowest (bacterial means of 0.999 and 0.902, respectively; fungal means of 0.950 and 0.680, respectively). However, the observed species richness showed the order GM > PC > PG.

**TABLE 2:** Alpha diversity of soil bacterial and fungal communities in different treatments.

	Simpson	Pielou_e	Observed_species
**Bacterium**			
PC	0.999 ± 0.000	0.902 ± 0.003	6600.467 ± 80.759
GM	0.999 ± 0.000	0.905 ± 0.001	6721.933 ± 153.510
PG	0.999 ± 0.000	0.908 ± 0.001	6340.367 ± 127.390
*p-*value	0.18	0.15	0.18
**Fungus**			
PC	0.950 ± 0.021	0.680 ± 0.030	478.900 ± 15.459
GM	0.973 ± 0.004	0.746 ± 0.017	629.600 ± 52.620
PG	0.977 ± 0.010	0.772 ± 0.026	477.133 ± 45.252
*p-*value	0.39	0.11	0.12

The data in the table for the mean standard ± error. PC, poplar single cropping; GM, black bean single cropping; PG, poplar black bean intercropping.

**FIGURE 1:**
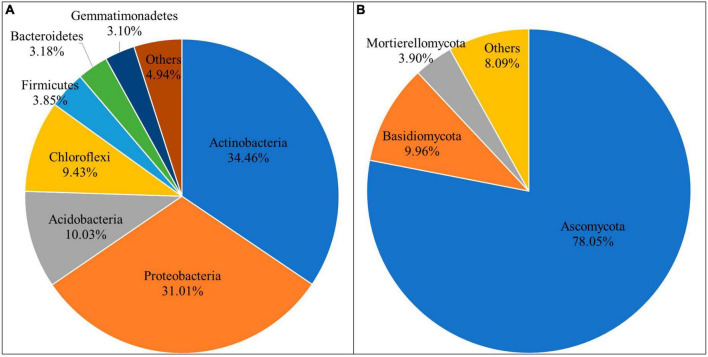
Relative abundance of dominant bacterial phyla **(A)** and fungal phyla **(B).**

Circle packing chart and pie chart were used to show the composition proportions of different taxonomic units of microbial communities in different groups. The results showed that there were differences in the abundance of bacterial and fungal communities at different microbial taxonomic levels (Supplementary Figures 2, 3). In the bacterial community, 39 phyla were detected in all samples (Supplementary Table 1). The dominant phyla were Actinobacteria, Proteobacteria, Acidobacteria, Chloroflexi, Firmicutes, Bacteroidetes, and Gemmatimonadetes. They accounted for 95.06% of the total population, respectively, 34.46, 31.01, 10.03, 9.43, 3.85, 3.18, 3.10% (Figure 1A). The remaining sequences belonged to 32 bacterial phyla such as Verrucomicrobia, Nitrospirae, and Rokubacteria, accounting for less than 1%.

A total of 11 phyla were detected in the fungal community (Supplementary Table 2). They were Ascomycota, Basidiomycota, Mortierellomycota, Glomeromycota, Zoopagomycotina, Chytridiomycota, Olpidiomycota, Basidiobolomycota, Aphelidiomycota, Rozellomycota, Mucoromycota, respectively (Figure 1B). Among them, only Ascomycota, Basidiomycota and Mortierellomycota had relative abundance higher than 1%, accounting for 78.05, 9.96, and 3.90%, respectively. Notably, Basidiobolomycota was not found in GM, while both Rozellomycota and Mucoromycota were found only in PG.

**FIGURE 2:**
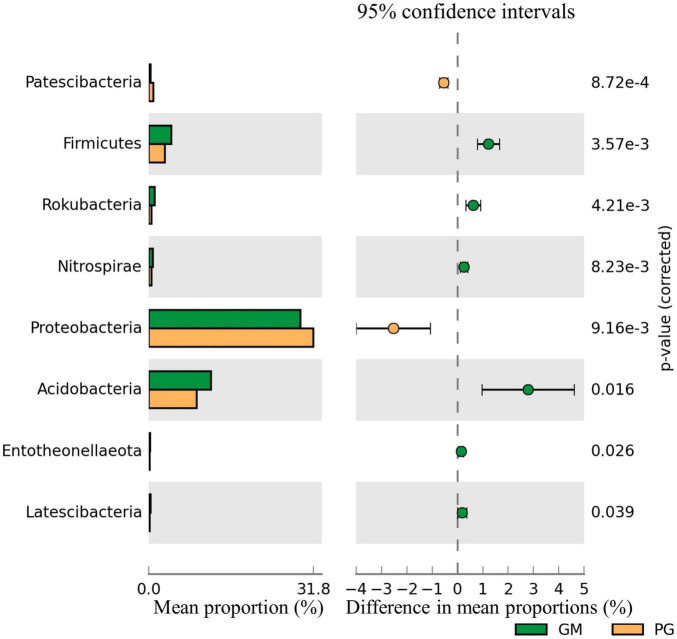
Difference of soil bacterial community of GM and PG. Only the phyla with significant differences in relative abundance under different treatments were shown in the figure (*p* < 0.05). GM, black bean single cropping; PG, poplar black bean intercropping.

**FIGURE 3:**
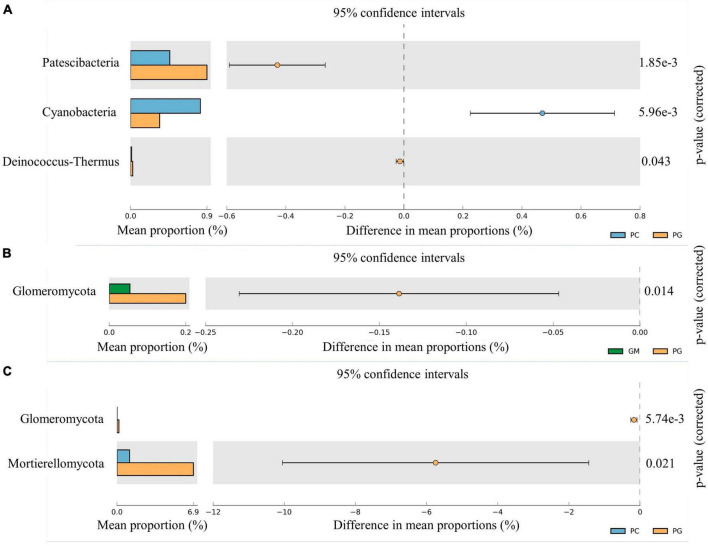
Microphyla difference of soil bacterial community under different treatments. Only the phyla with significant differences in relative abundance under different treatments were shown in the figure (*p* < 0.05). **(A)** Soil bacteria of PC and PG; **(B)**: soil fungi of GM and PG; **(C)**: soil fungi of PC and PG. PC, poplar single cropping; GM, black bean single cropping; PG, poplar black bean intercropping.

The relative abundance of Patescibacteria and Proteobacteria in the soil bacterial communities of PG was significantly higher than GM (*p* < 0.05). However, compared with GM, the relative abundance of Firmicutes, Rokubacteria, Nitrospirae, Acidobacteria, Entotheonellaeota and Latescibacteria of PG was significantly decreased (*p* < 0.05) (Figure 2). However, the relative abundance of only Glomeromycota in the fungal communities differed significantly between the two groups, as PG > GM (Figure 3B). In the soil bacterial communities of PG, compared with PC, only the relative abundances of Patescibacteria, Cyanobacteria and Deinococcus-Thermus were found to be significantly different (*p* < 0.05) (Figure 3A). Among them, the relative abundance of Cyanobacteria was higher in PC than in PG, and that of Patescibacteria and Deinococcus-Thermus was higher in PG than in PC. There were two phyla in the fungal communities with significant differences in relative abundance, and PG was higher than PC, respectively: Glomeromycota and Mortierellomycota (Figure 3C).

**FIGURE 4:**
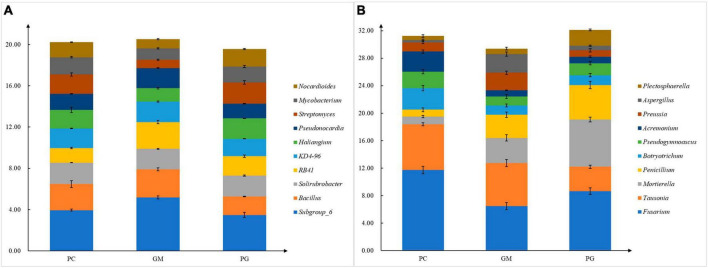
Different compositions of soil bacterial **(A)** and fungal **(B)** genera under different treatments. PC, poplar single cropping; GM, black bean cropping: PG, poplar black bean intercropping.

There were also differences in soil microorganisms in different treatments at the genus level, and only the bacteria genera with the top ten relative abundance were analyzed. In the bacterial community, *Subgroup_6*, *Bacillus*, *KD4-96*, and *Pseudonocardia* had the highest relative abundance in GM (5.18, 2.72, 1.99, 1.96%). The relative abundance of them in PG were the lowest (3.65, 1.72, 1.67, 1.41%) (Figure 4A). The relative abundance of *Haliangium* and *Nocardioides* was the highest in PG and the lowest in GM. The relative abundances of the remaining four genera of PG were located between PC and GM. In the fungal community, the top ten relative abundance genera were *Fusarium*, *Tausonia*, *Mortierella*, *Penicillium*, *Plectosphaerella*, *Botryotrichum*, *Pseudogymnoascus*, *Acremonium*, and *Preussia* (Figure 4B). *Mortierella*, *Penicillium*, and *Plectosphaerella* had the highest relative abundance in PG, while *Tausonia* and *Preussia* had the lowest relative abundance in PG.

**FIGURE 5:**
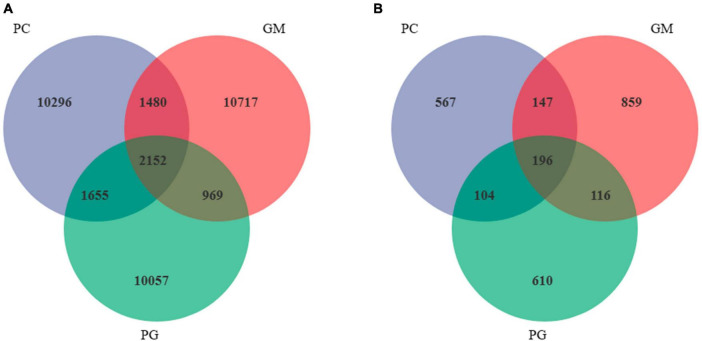
I Venn diagram showing the shared bacterial **(A)** and fungal **(B)** ASVs in all treatments. PC, poplar single cropping; GM, black bean single cropping; PG: poplar black bean intercropping.

A total of 37,326 bacterial ASVs were detected in the soil of the three samples, among which PC, GM, and PG had 15,583, 15,318, and 14,833 ASVs, respectively (Figure 5A). There were 2,152 ASVs shared by the three groups. PC had the most unique ASV of 10,296, while PG had the least unique ASV of 10,057. In addition, a total of 2,599 fungal ASVs were detected, including 1,014, 1,318 and 1,026 ASVs for PC, GM, and PG, respectively (Figure 5B). The three have 196 ASVs in common. The largest number of GM-specific ASVs were 859, while the number of PC-specific ASVs was only 567.

**FIGURE 6:**
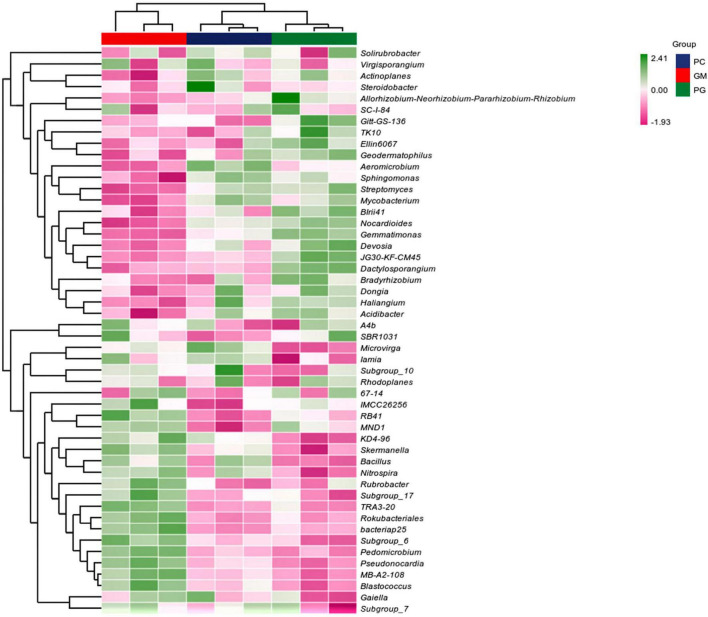
Based on the average algorithm, the clustering heat maps of soil bacteria with the relative abundance of top 50 were performed at the genus level. PC, poplar single cropping; GM, black bean single cropping; PG, poplar black bean intercropping.

**FIGURE 7:**
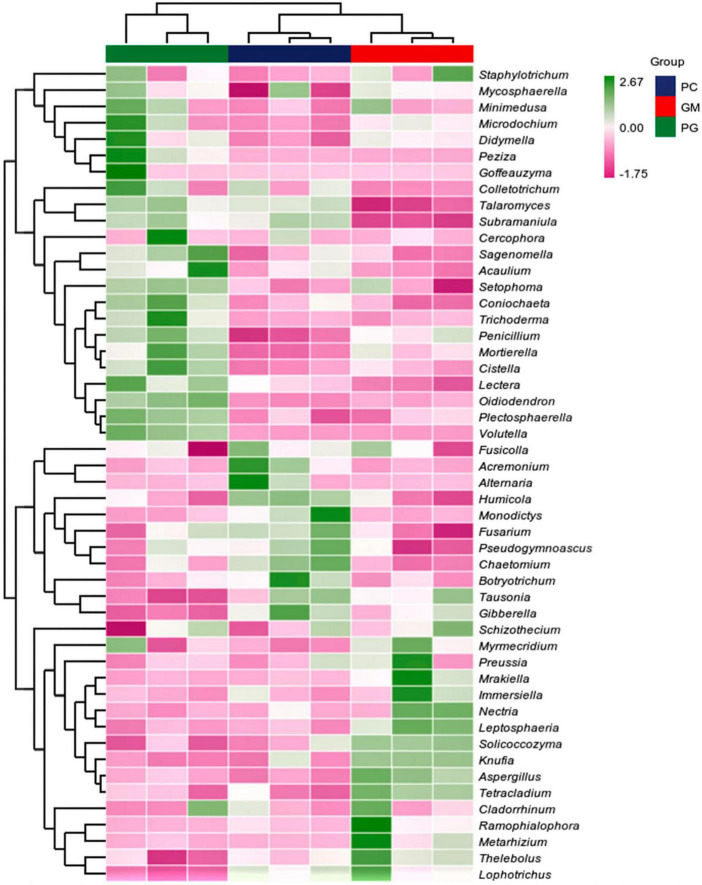
Based on the average algorithm, the clustering heat maps of soil fungi with the relative abundance of top 50 were performed at the genus level. PC, poplar single cropping; GM, black bean single cropping; PG, poplar black bean intercropping.

In the soil bacterial community, the maximum number of ASVs shared by PC and PG was 3807, indicating that the soil bacterial communities of PC and PG were more similar (Figure 5A). It could also be seen from Figure 6 that at the genus level, PC and PG were clustered into one class and then aggregated with GM. This also indicated that the soil bacterial communities of PC and PG were more similar. In the soil fungal community, PC and GM shared the most ASVs (Figure 5B). In the heatmap, PC and GM were grouped together and then aggregated with PG, indicating that the soil fungal communities between PC and GM were more similar (Figure 7).

### Correlations between soil properties and soil dominant microphyla and microbial communities

Soil chemistry was closely related to the relative abundance of dominant phyla (Table 3). Soil pH value was significantly correlated with the relative abundance of Gemmatimonadetes and Mortierellomycota (*r* = −0.708, *p* < 0.05; *r* = −0.779, *p* < 0.05). The contents of total carbon and total nitrogen and carbon nitrogen ratio in soil were significantly negatively correlated with the relative abundance of Proteobacteria (*r* = −0.790, *p* < 0.05; *r* = −0.683, *p* < 0.05; *r* = −0.738, *p* < 0.05), which was significantly positively correlated with the relative abundance of Acidobacteria (*r* = 0.890, *p* < 0.01; *r* = 0.788, *p* < 0.05; *r* = 0.771, *p* < 0.05). Soil total phosphorus content was significantly correlated with the relative abundance of Chloroflexi and Gemmatimonadetes (*r* = 0.914, *p* < 0.01; *r* = 0.710, *p* < 0.05). The content of soil available phosphorus significantly associated only with Mortierellomycota relative abundance (*r* = 0.869, *p* < 0.01). The relative abundance of Firmicutes was only significantly correlated with soil C/N (*r* = 0.784, *p* < 0.05).

**TABLE 3:** Person correlation between soil properties and relative abundance of bacterial and fungal phylum.

Phylum		pH	Total carbon	Total nitrogen	Total phosphorus	Available phosphorus	Carbon nitrogen ratio
Bacterium	Actinobacteria	0.374	−0.612	−0.589	−0.443	−0.140	−0.438
	Proteobacteria	0.184	−0.790*	−0.683*	−0.310	0.312	−0.738*
	Acidobacteria	−0.283	0.890**	0.788*	0.448	−0.106	0.771*
	Chloroflexi	−0.416	0.343	0.532	0.914**	0.482	−0.094
	Firmicutes	0.251	0.417	0.152	−0.406	−0.664	0.784*
	Bacteroidetes	0.530	−0.617	−0.617	−0.453	−0.152	−0.407
	Gemmatimonadetes	−0.708*	0.289	0.418	0.710*	0.493	−0.006
Fungus	Ascomycota	0.565	−0.079	−0.249	−0.562	−0.582	0.220
	Basidiomycota	−0.375	0.148	0.279	0.575	0.386	−0.083
	Mortierellomycota	−0.779*	−0.004	0.191	0.633	0.869**	−0.387

*Indicates *p* < 0.05.

**Indicates *p* < 0.01.

**FIGURE 8:**
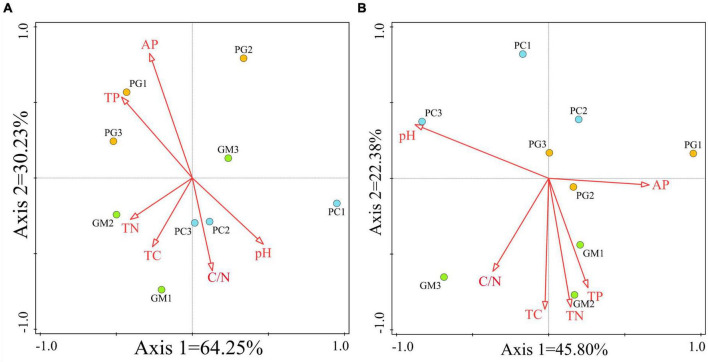
Redundancy analysis (RDA) of soil chemical properties and soil bacterial **(A)** and fungal **(B)** communities was performed based on ASV levels. TC, total carbon; TN, total nitrogen; TP, total phosphorus; AP, available phosphorus; PC, poplar single cropping; GM, black bean single cropping; PG, poplar black bean intercropping.

RDA analysis was used to analyze the relationship between soil microbial community alpha diversity and soil chemical properties (Figure 8). In the bacterial community, the total variation of RDA was 27, and the explanatory variables account for 97.1%, of which the differences in the first two axes accounted for 94.48% (Figure 8A). The arrows of available phosphorus and total phosphorus were the longest, indicating that the content of total phosphorus and available phosphorus in soil was the main factor affecting the diversity of bacterial community, followed by pH and carbon nitrogen ratio, and total nitrogen and total carbon were the shortest. In the fungal community, the total variation of RDA was 27, and the explanatory variables account for 69.8%, of which the differences in the first two axes accounted for 68.18% (Figure 8B). pH had the longest arrow, indicating that soil pH was the main factor affecting fungal community diversity, followed by total carbon, total nitrogen and carbon nitrogen ratio, but available phosphorus was the least influential factor among the six factors.

## Discussion

Poplar and black bean intercropping significantly increased the amount of soil available phosphorus content when compared to single cropping, proving that intercropping promotes efficient use of soil nutrients. This hypothesis was also supported by Zhang et al. (2018). The increased prevalence of related bacteria, such as rhizobium, phototrophic bacteria, and potassium-trophic bacteria, which are involved in the soil nutrient cycle, could be one explanation (Zhang et al., 2018). Compared with poplar single cropping, intercropping significantly increased the content of total phosphorus in soil, and significantly decreased soil carbon nitrogen ratio. Additionally, although the effect was not significant, the pH of the soil decreased and the total carbon and nitrogen content of the soil increased. Among the legume plants is the black bean. The symbiotic nitrogen fixation of legume crops is the accumulation of nitrogen for plant growth through the symbiotic nitrogen fixation with rhizobia (Zhong et al., 2023). This may be the main reason for the increase of soil total nitrogen content after intercropping. Soil acidification was one of the direct responses to the increase of soil nitrogen content (Liu et al., 2006, 2013), which also explained the reason for the decrease of soil pH value in intercropping. In addition, studies have shown that the increase of soil nitrogen content can lead to the increase of soil total carbon (Yuan et al., 2020). However, compared with poplar single cropping, the change of soil total carbon and nitrogen content in intercropping was not significant, which may be due to the limited nitrogen fixation ability of herbaceous plants, and the effect of short-term nitrogen fixation on soil nutrients was not significant (Yang et al., 2021).

Simpson index and Pielou’s evenness index both showed that the diversity and evenness of microbial communities (bacteria and fungi) in intercropped soils were higher than those in monoculture soils, although this was not significant. This is due to tree-based intercropping systems that exhibit more heterogeneous patterns of vegetation cover and rooting, thereby increasing the diversity of soil microbial communities (Lacombe et al., 2009). Actinobacteria, Proteobacteria, Acidobacteria, Chloroflexi, Firmicutes, Bacteroidetes, and Gemmatimonadetes were the most prevalent phyla of bacteria in each sample. Three phyla of fungi, Ascomycota, Basidiomycota and Mortierellomycota, were the most prevalent. According to Caporaso et al. (2012) and Tedersoo et al. (2017), these were all common phyla found in soil. Numerous studies have demonstrated that the interaction between vegetation species richness and soil use patterns may affect microbial community structure (Chung et al., 2007; Jangid et al., 2011). The relative abundance of soil microorganisms varied between intercropping and monoculture at the phylum and genus levels, suggesting that planting practices altered the makeup of the soil microbial community.

At the bacterial level, Acidobacteria was considered to be an oligotrophic taxon and Proteobacteria was considered to be a syntrophic taxon (Yan et al., 2020; Liang et al., 2021). This also explained why, in our study, these two phyla had completely opposite correlations with soil nutrients. RDA analysis showed that pH was the main factor affecting soil fungal community diversity. Previous studies have shown that changes in soil microbial communities were closely related to soil pH value (Lauber et al., 2009; Wan et al., 2020). Total phosphorus and available phosphorus are the main factors affecting soil bacterial community diversity, which is consistent with previous findings of Zhang et al. (2016) that soil available phosphorus is closely related to bacterial community structure and diversity.

*Bradyrhizobium* and *Allorhizobium-Neorhizobium-Pararhizobium-Rhizobium* associated with nitrogen fixation were found in the top 50 relative abundance at the bacterial genus level (Figure 6). Intercropping increased their relative abundance in the soil. This is possible because intercropping can enhance the nitrogen fixation capacity of legumes when non-legumes are strong competitors for soil inorganic nitrogen (Neugschwandtner and Kaul, 2015; Jin et al., 2023). In our work, we found *Bacillus*, *Bradyrhizobium*, *Fusarium*, *Penicillium*, and *Aspergillus*, which have been identified in recent studies as phosphorus-solubilizing bacteria and fungi (Zhang et al., 2016; Zhou et al., 2023). And they can help plants absorb phosphorus (Kalayu, 2019). Compared with poplar monoculture, intercropping increased the relative abundance of *Bradyrhizobium*, *Penicillium*, and *Aspergillus*, and decreased the relative abundance of *Bacillus* and *Fusarium*. Multiple results showed that there were differences in the composition of soil microbial communities between intercropping and monocropping.

## Conclusion

In conclusion, intercropping poplar and black bean had a considerable impact on the nutrients in the soil as well as the makeup of the microbial community (bacteria and fungi) composition, but not on the diversity of the community. When intercropping was used instead of poplar single cropping, the soil’s total phosphorus content increased and the ratio of soil carbon to nitrogen significantly decreased. Additionally, although the effect was not significant, the pH of the soil decreased and the total carbon and nitrogen content of the soil increased. Intercropping reduced community richness while increasing the soil microbial community’s evenness and diversity within its habitat. When it came to the makeup of the microbial communities, intercropping and poplar single cropping were more similar in terms of the composition of the bacterial and fungal communities, respectively, but they were less similar in terms of the composition of the fungal communities. The relative abundance of soil-dominant bacteria is correlated with the nutrient content of the soil, according to the person correlation analysis. Proteobacteria, Acidobacteria, Chloroflexi, Firmicutes, Gemmatimonadetes and Mortierellomycota exhibited significant correlations (*p* < 0.05) with various soil nutrient factors. Furthermore, soil pH had the biggest impact on the diversity of the soil fungal community, whereas soil total phosphorus and available phosphorus content had the greatest effects on the diversity of the soil bacterial community. This study can serve as a theoretical foundation for the development of a poplar and black bean-based forest-agricultural complex management system in the future.

## Data availability statement

The datasets presented in this study can be found in online repositories. The names of the repository/repositories and accession number(s) can be found in the article/Supplementary material.

## Author contributions

JLi: Data curation, Methodology, Writing – original draft. WxiZ: Data curation, Writing – original draft. CT: Investigation, Writing – original draft. ZP: Investigation, Writing – original draft. YP: Investigation, Writing – original draft. JQ: Formal Analysis, Writing – original draft. JLe: Methodology, Writing – original draft. XS: Project administration, Writing – original draft, Writing – review and editing. WxuZ: Project administration, Writing – review and editing. CD: Funding acquisition, Resources, Writing – review and editing.

## Footnotes

1
https://docs.qiime2.org/2019.4/tutorials/
