# Supplementary material for: Intercropping changed the soil microbial community composition but no significant effect on alpha diversity
Source: Front Microbiol. 2024 Mar 20;15:1370996. doi: 10.3389/fmicb.2024.1370996 (PMC10988756; doi:10.3389/fmicb.2024.1370996)
Supplement: Supplementary file 1 [file Table_1.docx]

**Supplementary Table 1. Relative abundance (%) of bacterial phyla under different treatments.**

|  | **PC** | **GM** | **PG** |
| --- | --- | --- | --- |
| Actinobacteria | 35.206327 | 33.281582 | 34.8966 |
| Proteobacteria | 32.024013 | 29.22731 | 31.77281 |
| Acidobacteria | 8.857565 | 12.011987 | 9.230983 |
| Chloroflexi | 8.559361 | 9.745029 | 9.993088 |
| Firmicutes | 4.137555 | 4.314187 | 3.08889 |
| Bacteroidetes | 3.701602 | 2.704977 | 3.131319 |
| Gemmatimonadetes | 2.928431 | 3.15206 | 3.216067 |
| Verrucomicrobia | 0.748684 | 0.800966 | 0.693275 |
| Nitrospirae | 0.723052 | 0.816132 | 0.555956 |
| Rokubacteria | 0.401041 | 1.108051 | 0.481045 |
| Planctomycetes | 0.409045 | 0.838254 | 0.528875 |
| Patescibacteria | 0.450063 | 0.337515 | 0.878235 |
| Cyanobacteria | 0.802215 | 0.303865 | 0.334186 |
| Latescibacteria | 0.189212 | 0.346003 | 0.155689 |
| Entotheonellaeota | 0.100164 | 0.266337 | 0.122819 |
| Armatimonadetes | 0.125507 | 0.089223 | 0.160374 |
| Elusimicrobia | 0.110713 | 0.104003 | 0.13728 |
| Chlamydiae | 0.076562 | 0.066284 | 0.127383 |
| Fibrobacteres | 0.057552 | 0.048572 | 0.067055 |
| WPS-2 | 0.040825 | 0.027231 | 0.056221 |
| Dependentiae | 0.036723 | 0.03424 | 0.04905 |
| Omnitrophicaeota | 0.014553 | 0.046087 | 0.018846 |
| Spirochaetes | 0.015066 | 0.023512 | 0.023908 |
| Fusobacteria | 0.02141 | 0.009287 | 0.030989 |
| Deinococcus-Thermus | 0.011297 | 0.009854 | 0.024747 |
| BRC1 | 0.012672 | 0.004989 | 0.01751 |
| GAL15 | 0.008405 | 0.015885 | 0.0041 |
| FBP | 0.00437 | 0.011543 | 0.006502 |
| WS2 | 0.008872 | 0.002629 | 0.009426 |
| FCPU426 | 0 | 0.003514 | 0.004416 |
| Dadabacteria | 0 | 0.006711 | 0 |
| Zixibacteria | 0 | 0.006601 | 0 |
| Kiritimatiellaeota | 0.001667 | 0.001769 | 0.00122 |
| Hydrogenedentes | 0 | 0.002652 | 0.000814 |
| Synergistetes | 0.002001 | 0 | 0.001239 |
| Margulisbacteria | 0.000891 | 0 | 0.001201 |
| Tenericutes | 0.001337 | 0 | 0 |
| Epsilonbacteraeota | 0 | 0.001327 | 0 |
| WS4 | 0 | 0.000884 | 0 |
| Others | 0.211245 | 0.228946 | 0.177887 |

PC: poplar single cropping; GM: black bean single cropping; PG: poplar black bean intercropping.

**Supplementary Table 2. Relative abundance (%) of fungal phyla under different treatments.**

|  | **PC** | **GM** | **PG** |
| --- | --- | --- | --- |
| Ascomycota | 84.53953 | 83.37012 | 73.5987 |
| Basidiomycota | 6.895749 | 7.778079 | 12.17501 |
| Mortierellomycota | 1.111858 | 2.796996 | 6.64974 |
| Glomeromycota | 0.010136 | 0.035752 | 0.204727 |
| Zoopagomycota | 0.006454 | 0.073957 | 0.050509 |
| Chytridiomycota | 0.005046 | 0.096482 | 0.012744 |
| Olpidiomycota | 0.001083 | 0.030961 | 0.033703 |
| Basidiobolomycota | 0.021968 | 0 | 0.019333 |
| Aphelidiomycota | 0.0064 | 0.013409 | 0.021245 |
| Rozellomycota | 0 | 0 | 0.038553 |
| Mucoromycota | 0 | 0 | 0.001683 |
| Others | 7.401774 | 5.804244 | 7.194055 |

PC: poplar single cropping; GM: black bean single cropping; PG: poplar black bean intercropping.
