# Supplementary material for: Intercropping changed the soil microbial community composition but no significant effect on alpha diversity
Source: Front Microbiol. 2024 Mar 20;15:1370996. doi: 10.3389/fmicb.2024.1370996 (PMC10988756; doi:10.3389/fmicb.2024.1370996)
Supplement: Supplementary file 2 [file Data_Sheet_1.docx]

Supplementary Material


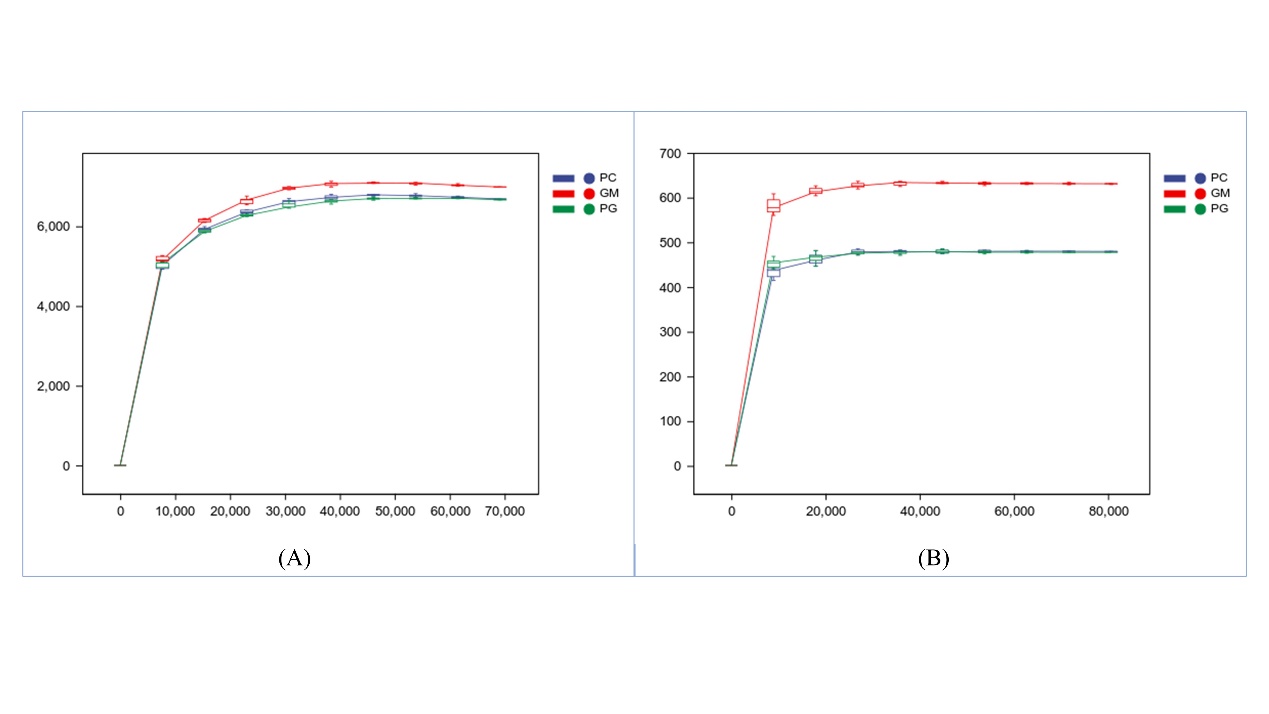


**Supplementary Figure 1.** Sparse curve of soil bacterial (A) and fungal (B) communities. PC: poplar single cropping; GM: black bean single cropping; PG: poplar black bean intercropping.


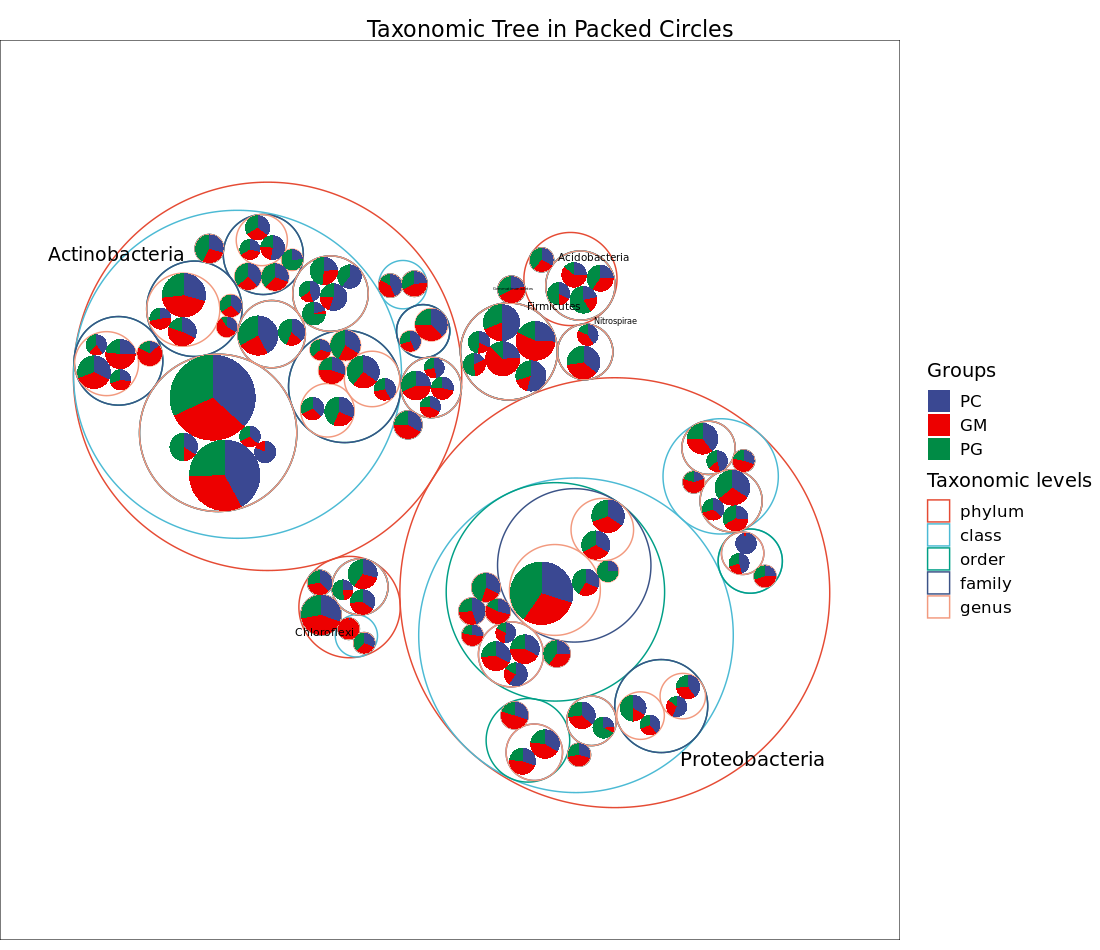


**Supplementary Figure 2.** Taxonomic tree in packed circles of soil bacterial communities in different samples. PC: poplar single cropping; GM: black bean single cropping; PG: poplar black bean intercropping.

**
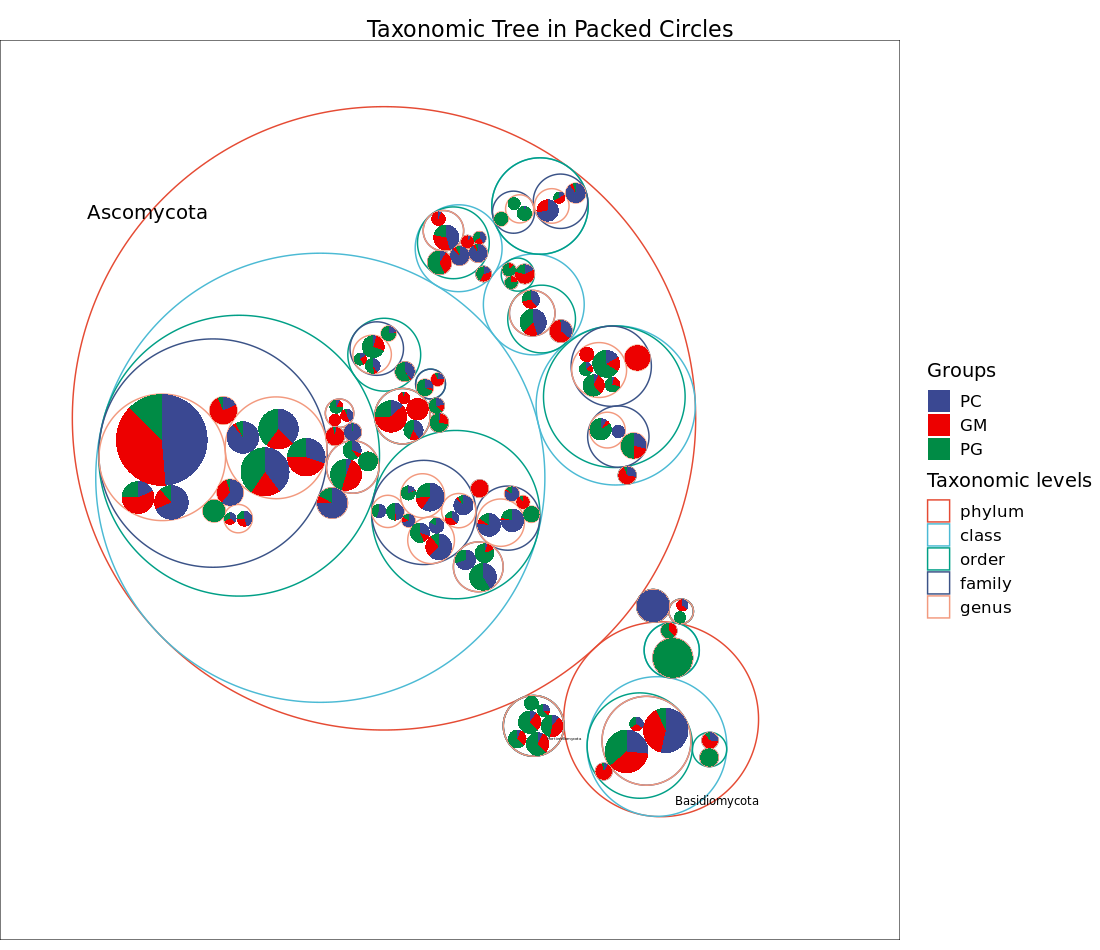
**

**Supplementary Figure 3.** Taxonomic tree in packed circles of soil fungal communities in different samples. PC: poplar single cropping; GM: black bean single cropping; PG: poplar black bean intercropping.
